# A Importância da Radiação de Referência

**DOI:** 10.36660/abc.20200590

**Published:** 2020-12-01

**Authors:** Luiz Alberto Alberto Christiani

**Affiliations:** 1 Universidade do Estado do Rio de Janeiro Rio de JaneiroRJ Brasil Universidade do Estado do Rio de Janeiro, Rio de Janeiro, RJ – Brasil

**Keywords:** Cateterismo Cardíaco/métodos, Cardiopatias Congênitas, Exposição à Radiação, Criança

Todo mundo sabe a importância das referências para avaliar a velocidade do processador de um computador. Também podemos obter alguma noção a partir do próprio computador, quando apresenta lentidão ou quando não consegue abrir um novo programa. Portanto, devemos buscar os melhores e mais rápidos processadores para resolver o problema. A empresa responsável por fazer esses chips de computador também é aquela que cria as referências de mercado para novos processadores, e precisamos dessas informações para cada decisão que tomamos ao comprar ou atualizar um computador.

Referência está presente em várias situações, sempre que precisamos comparar como estamos trabalhando. E quando chega uma nova proposta, quais seriam as melhores práticas.

Quando trabalhamos na sala de hemodinâmica, devemos saber até onde vai a radiação para que possamos lidar com o paciente de forma segura e, mais importante, se mudarmos um protocolo, temos que fazer uma escolha sensata.

Por muitos anos, nosso objetivo na sala de hemodinâmica foi obter uma imagem perfeita e fazer um diagnóstico completo da cardiopatia para encaminhar o paciente para cirurgia. Isso foi em outro século, e outra forma de pensar a Cardiologia Pediátrica e como tratar as cardiopatias congênitas. Outras ferramentas diagnósticas começaram a ser utilizadas, como o ecocardiograma e a tomografia. Naquela época, as preocupações com a radiação eram bem menores! No laboratório, “novas máquinas” (hoje adequadas apenas para a história) foram forçadas a colocar muitas imagens em um rolo de filme para alcançar o que estava “escondido” no coração de uma criança. Assim como Marie Curie descobriu o rádio e muitos anos depois morreu pelas consequências de seu grande trabalho, nós simplesmente empregamos a radiação sem “ver” o que estava além dela.

A média global da dose efetiva anual de radiação (considerando a susceptibilidade de prejudicar diferentes órgãos) por pessoa é cerca de 2,4 mSv (Sievert) e varia de 1 a mais de 10 mSv dependendo de onde as pessoas vivem (cerca de 6 mSv nos EUA). A maioria (80%) vem de fontes naturais. A exposição clínica é responsável por 98 por cento da exposição à radiação de todas as fontes artificiais e é o segundo maior fator para a exposição da população em todo o mundo.^1^

Na Cardiologia Pediátrica, isso pode ser mais importante. A radiografia convencional em crianças cardiopatas representa 92% dos exames, enquanto o cateterismo cardíaco representa 1,5%. Já os exames de cateterismo contribuíram com 60% da exposição cumulativa e, se somados à tomografia computadorizada, representaram 81% da exposição cumulativa.^2^ O risco associado à exposição à radiação é particularmente relevante para crianças com doenças cardíacas mais complexas, que frequentemente recebem imagens repetitivas com modalidades de alta exposição.^2^

Hoje, como temos outras ferramentas para explorar o coração, como o ecocardiograma, a tomografia ou a ressonância magnética, podemos antecipar as informações mais importantes de que precisamos. A sala de hemodinâmica agora se dedica principalmente a procedimentos terapêuticos. Por isso, quem lida com cardiopatias congênitas deve ter um conhecimento completo de cada doença a ser estudada ou tratada. Nunca devemos ser um “fechador de buracos” de comunicações interatriais ou de comunicações interventriculares.

Muitos artigos de diversos centros médicos importantes chamaram a atenção para o problema da radiação no cateterismo e como reduzi-la a níveis inferiores. As crianças são especialmente vulneráveis à radiação, como aponta o artigo de Manica et al.^3^ Elas têm maior superfície cutânea e geralmente maior área exposta à radiação nos exames. Na maioria dos centros médicos de nosso país que trabalham com exames de cardiopatia congênita e cateterismo, a radiação não é medida e controlada de forma adequada.

O artigo de Manica et al.,^3^ é um estudo muito importante e enfatiza a necessidade de uma medição simples e útil para o controle da radiação em laboratório. A dose efetiva de radiação é uma variável de cálculo complexo e absolutamente impraticável. Por outro lado, o DAP ( *dose area product* ou produto dose-área) é automaticamente visível pelo equipamento e, conforme apontado por Kobayashi et al.,^4^e sugerido neste artigo, o DAP/m2 pode ser usado em crianças como uma boa referência a ser aplicada no mesmo laboratório para comparar diferentes períodos com modificações no nível de radiação — e para que cada laboratório saiba se os protocolos em uso são adequados ou não. Os autores também demonstram um detalhe muito importante que o profissional vê no dia a dia do trabalho: os exames diagnósticos podem ser mais demorados do que o tratamento,^3^ liberam uma quantidade maior de radiação no paciente e posteriormente na equipe envolvida.

Como esperávamos, o “produto fluoroscópico de peso” teve uma boa correlação com o DAP. Portanto, mesmo que não se saiba nada sobre o DAP, deve-se ter cuidado em relação a por quanto tempo “colocar os pés” na imagem fluoroscópica e, mais importante, deve-se usar uma taxa de quadros baixa e uma dose tão mínima quanto possível. Conforme apontado por Borik et al.,^5^ modificações simples podem representar uma redução significativa da dose, como a taxa de quadros de fluoroscopia de 7,5 quadros/segundo, utilizando a “técnica de *air gap* ”^6^ e uma ampliação mínima, com o detector posicionado o mais longe possível da criança.

Em outro estudo recente com exposição controlada à radiação feito por Hill et al.,^7^ confirma-se que modificações simples e essenciais devem ser utilizadas na prática cotidiana. Dados apresentados por Borik et al.,^5^ e Cevallos et al.,^8^ incluem muitos pacientes estudados e organizam tabelas práticas com os procedimentos mais comuns e o respectivo DAP/kg.

Em nossa prática atual, mais exames com angiografia rotacional 3D são feitos e constituem um método essencial. Eles fornecem um roteiro em tempo real para procedimentos guiados por anatomia e diagnósticos mais precisos em algumas circunstâncias. No entanto, a quantidade de radiação pode ser alta se os protocolos não forem implementados. Minderhoud et al.,^9^ demonstraram que uma simples modificação do protocolo pode reduzir a exposição de todo o cateterismo.

Portanto, o trabalho de Manica et al.,^3^agrega uma ferramenta muito importante para o controle da radiação em nossa prática cotidiana: o DAP/kg. Como uma referência simples e eficaz para a radiação no cateterismo de cardiopatias congênitas, deve ser incluída em todos os laudos laboratoriais.

## References

[1] . United Nations Environment Programme. Radiation: effects and sources. USA;2016.

[2] . Johnson J, Hornick P, Li J.Cumulative radiation exposure and cancer risk estimation in children with heart disease”, Circulation. 2014;130(2):161-7. 10.1161/CIRCULATIONAHA.113.005425 PMC4103421 24914037

[3] . Manica JL, Duarte VO, Ribeiro M, Hartley A, Petraco R, Pedra C, et al. Standardizing Radiation Exposure During Cardiac Catheterization In Children With Congenital Heart Disease: Data From A Multicenter Brazilian Registry. Arq Bras Cardiol. 2020; 115(6):1154-1161. 10.36660/abc.20190012 PMC8133734 33084745

[4] . Kobayashi D, Meadows J, Forbes T. “Standardizing radiation dose reporting in the pediatric cardiac catheterization laboratory - A multicenter study by the CCISC (congenital cardiovascular interventional study consortium), Cath Card Interv.2014;84(5):785-93. 10.1002/ccd.25467 24585540

[5] . S. Borik S, Devadas S, Mroczek D, BIOMED-EnG, Kiang L, Chatuverdi R, et al. Achievable radiation reduction during pediatric cardiac catheterization: How low can we go?”, Catheter Cardiovasc Interv.2015;86(5): 841-8. 10.1002/ccd.26024 26011560

[6] . Partridge J, McGahan G, Causton S, Bowers M, Mason M, Dalby M, et al. Radiation dose reduction without compromise of image quality in cardiac angiography and intervention with the use of a flat panel detector without an antiscatter grid, Heart.2006;92(4):507-10. 10.1136/hrt.2005.063909 PMC1860862 16159965

[7] . Hill D, Wang C, Einstein AJ, Januzis N, Nguyen G, Li JS, et al. Impact of imaging approach on radiation dose and associated cancer risk in children undergoing cardiac catheterization. , Catheter Cardiovasc Interv.2017;89(5):888-97. 10.1002/ccd.26630 PMC5164876 27315598

[8] . Cevallos C, Armstrong A, Glatz A, Goldstein BH, Gudausky TM, Leahy RA, et al. Radiation dose benchmarks in pediatric cardiac catheterization: A prospective multi-center C3PO-QI study. Catheter Cardiovasc Interv. 2017;90(2):269-80. 10.1002/ccd.26911 28198573

[9] . Minderhoud S, van der Stelt F, Molenschot M, Koster M, Krings GJ, Breur JMP. Dramatic Dose Reduction in Three-Dimensional Rotational Angiography After Implementation of a Simple Dose Reduction Protocol. Pediatr Cardiol, 2018;39(8):1635-41. 10.1007/s00246-018-1943-3 PMC6244991 30076424

