# Supplementary material for: A Importância da Radiação de Referência
Source: Arq Bras Cardiol. 2020 Dec 1;115(6):1162–3. [Article in Portuguese] doi: 10.36660/abc.20200590 (PMC8133725; doi:10.36660/abc.20200590)
Supplement: Translation

# Translation: The Importance of Benchmark Radiation

**Authors:** Luiz Alberto Alberto Christiani

**Affiliations:** ,1 Brazil Universidade do Estado do Rio de Janeiro Rio de Janeiro, RJ – Brazil

**Keywords:** Cardiac Catheterization/methods, Heart Defects Congenital, Radiation Exposure, Child

Everyone knows the importance of benchmark to evaluate processor speed in a computer. We can also have some inputs from the computer itself, as it goes slowly or when it is not able to open a new program. Therefore, we must pursue the best and the fastest processors to solve the problem. The company responsible for making those computer chips is also the one that creates new processor benchmarks, and we need that information for every decision we make when buying or upgrade a computer.

Benchmark is present in several situations, whenever we need to compare how we are working. And when a new proposal arrives, what would be the best practices.

When we work in the catheterization laboratory, we must know how far the radiation goes in order to offer safe patient management, and more importantly, if we change a protocol, we have to make a reasonable choice for it.

For many years, our goal in the catheterization laboratory was to achieve a perfect image and build a complete diagnosis of the heart defect in order to refer the patient to surgery. That was another century, and another way of thinking about Pediatric Cardiology and how to treat congenital heart diseases. Other diagnostic tools were beginning to be used, such as the echocardiogram and tomography. At that time, radiation concerns were minor! In the laboratory, “new machines” (nowadays only suitable for history) were forced to put many images in a roll film to achieve what was “hidden” in the small child’s heart. Just like Marie Curie discovering radium and many years later dying from the consequences of her great work, we simply employed radiation without “seeing” what was beyond that.

The global average annual effective dose of radiation (considering the susceptibility to harm different organs) per person is about 2.4 mSv (Sievert) and ranges from 1 to more than 10 mSv depending on where people live (about 6 mSv in the U.S.). The majority (80%) comes from natural sources. Medical exposure accounts for 98 percent of the radiation exposure from all artificial sources and is the second largest contributor to the population exposure worldwide.^1^

In Pediatric Cardiology, this can be more important. Conventional radiographs in children with heart disease represents 92% of examinations, whereas cardiac catheterization represents 1,5%. But catheterization exams contributed with 60% of cumulative exposure and, if added to computed tomography, they accounted for 81% of cumulative exposure.^2^The risk associated with radiation exposure is particularly relevant for children with more complex heart diseases, who often receive repetitive imaging with high-exposure modalities.^2^

Today, as we have other tools to explore the heart, like the echocardiogram, tomography or magnetic resonance imaging, we can anticipate the most important information we need. The catheterization laboratory is now mainly dedicated to therapeutic procedures. That is why those who deal with congenital heart diseases must have a complete knowledge of each patient disease to be studied or treated. We should never be a “hole closer” of atrial septal defects or ventricular septal defects.

Many articles from several important medical centers have drawn attention to the problem of radiation in catheterization, and how to bring it down to a lower level. Children are especially vulnerable to radiation, as pointed out by the article by Manica et al.^3^They have a greater skin surface and generally a bigger area exposed to radiation in exams. In most medical centers in our country that work with congenital heart disease and catheterizations exams, radiation is not adequately measured and controlled.

The article by Manica et al.^3^is a very important study, and it emphasizes the need of a simple and useful measurement for controlling radiation in the laboratory. The effective dose of radiation is a variable of complex calculation and it is absolutely not practical. On the other hand, DAP (Dose Area Product) is automatically visible by the machine and, as pointed out by Kobayashi et al.^4^and suggested in this article, DAP/m^2^can be used in children as a good benchmark to be applied in the same laboratory to compare different periods with radiation level modifications — and for every laboratory to know whether the protocols being used are adequate or not. The authors also demonstrate a very important detail that the practitioner sees every day at work: diagnostic exams can be more time consuming than therapeutics,^3^release a greater amount of radiation on the patient and on the staff involved afterwards.

As we have expected, the “weight-fluoroscopic product” had a good correlation with DAP. So even if you know nothing about DAP, you must be cautious about how long you “put your foot” on the fluoroscopic image, and more importantly, you must be sure to use a low frame rate and a dose as low as possible. As pointed out by Borik et al.,^5^simple modifications can represent a significant dose reduction, as the fluoroscopy frame rate of 7,5 frames/second, using the “air gap technique”^6^and a minimal magnification, with the detector placed as far as possible from children.

In another recent radiation exposure-controlled study by Hill et al.,^7^they confirm that simple and essential modifications must be used in everyday practice. Data presented by Borik et al.^5^and Cevallos et al.^8^include many patients studied and organized practical tables with the most common procedures and the respective DAP/kg.

In our current practice, more exams with 3D rotational angiography are done and constitute an essential method. They provide a real-time roadmap for anatomy-guided procedures and more precise diagnosis in some circumstances. However, the amount of radiation could be high if protocols are not implemented. Minderhoud et al.^9^demonstrated that a simple protocol modification can reduce the exposure of the entire catheterization exam.

Therefore, the work of Manica et al.^3^adds a very important tool to control radiation in our everyday practice: the DAP/kg. As a simple and effective benchmark for radiation in the catheterization of congenital heart disease, it should be included in every laboratory report.
